# Traditional processing unlocks anti-atherogenic potential of perilla fruit via PPAR-γ activation by luteolin

**DOI:** 10.1186/s40643-025-00957-7

**Published:** 2025-11-03

**Authors:** Yiqi Yan, Tian Xie, Ran An, Guangzhe Yao, Ming Huang, Ke Xiong

**Affiliations:** 1 Tianjin University of Traditional Chinese Medicine, Tianjin, 301617 China; 2 Institute of Traditional Chinese Medicine, Tianjin University of Traditional Chinese Medicine, Tianjin, 301617 China; 3 Haihe Laboratory of Modern Chinese Medicine, Tianjin, 301617 China; 4 State Key Laboratory of Component-Based Chinese Medicine, Tianjin University of Traditional Chinese Medicine, Tianjin, 301617 China; 5 School of Chinese Materia Medica, Tianjin University of Traditional Chinese Medicine, Tianjin, 301617 China; 6 Cangzhou Medical College, Cangzhou, 061001 China

**Keywords:** Perilla fruit, PPAR-γ, Traditional chinese medicine processing, Lipid regulation, Foam cell

## Abstract

The accumulation of foam cells, lipid-laden macrophages in atherosclerotic plaques, is a hallmark of cardiovascular disease progression. These cells contribute to chronic inflammation and plaque instability, underscoring the need for novel therapeutic strategies. Peroxisome proliferator-activated receptor gamma (PPAR-γ) is a nuclear receptor pivotal to lipid metabolism and inflammation control. Traditional Chinese Medicine (TCM) processing techniques, such as stir-frying, are believed to enhance herb efficacy, yet the molecular basis remains insufficiently understood. Here, we demonstrate that stir-fried Perilla fruit significantly increase luteolin content-a flavonoid compound capable of binding PPAR-γ at key residues (SER289, HIS323, PHE360, TYR473) as validated by molecular docking and dynamics simulation. In ox-LDL-induced RAW264.7 macrophages, luteolin promoted cholesterol efflux, reduced lipid accumulation, and upregulated PPAR-γ pathway proteins, effects that were abolished by the antagonist GW9662. These results provide mechanistic insight into the enhanced efficacy of stir-fried Perilla and highlight luteolin as a promising natural compound for atherosclerosis prevention.

**Graphical abstract:**

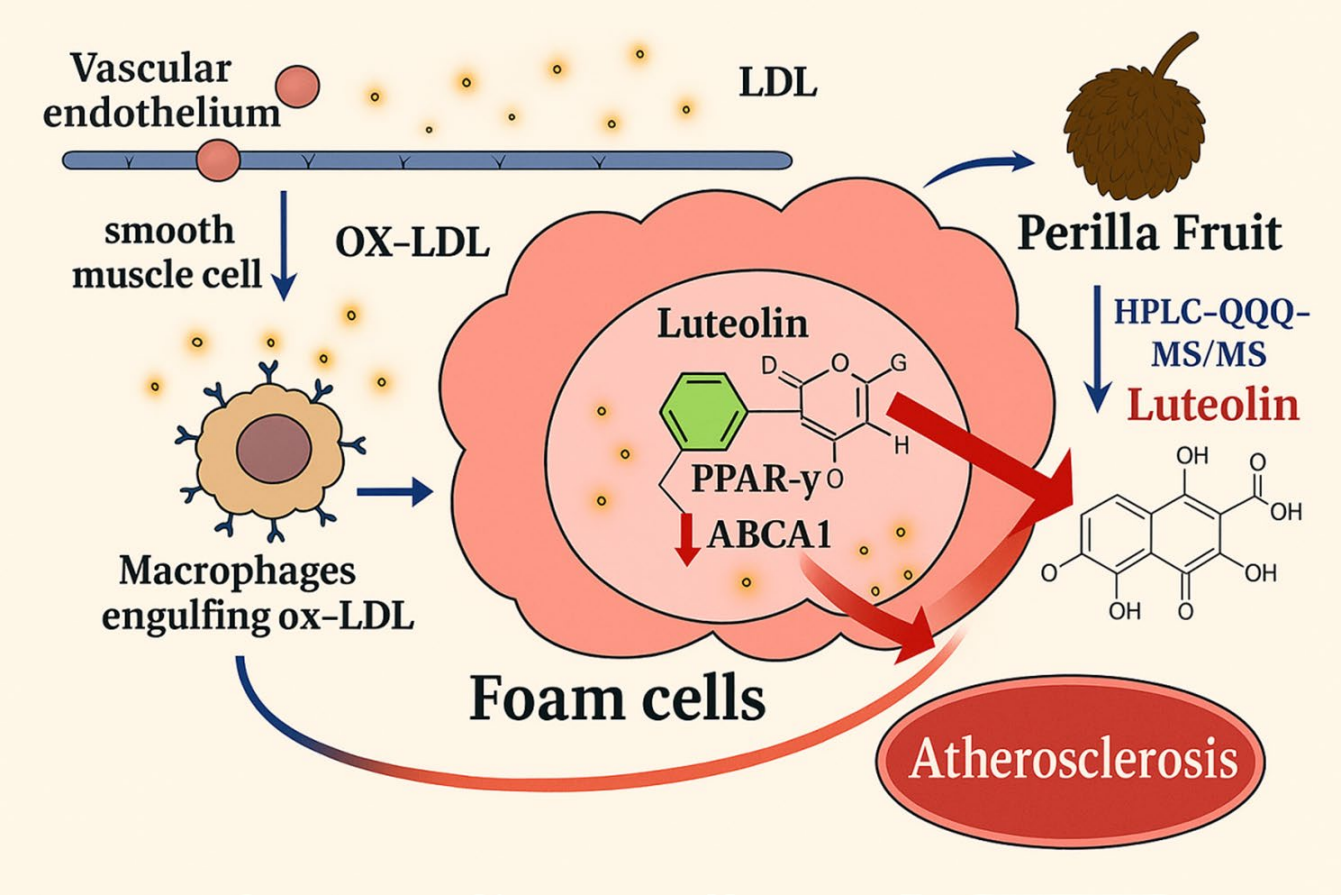

**Supplementary information:**

The online version contains supplementary material available at 10.1186/s40643-025-00957-7.

## Background

Foam cells are a hallmark of atherosclerotic plaques, and their formation is closely associated with multiple risk factors such as hyperlipidemia (Wang K et al. 2023), smoking (Pramanik et al. 2024), hypertension (Burger et al. 2021), and diabetes (Cervantes et al. 2023). Epidemiological data indicate that atherosclerosis is a primary cause of cardiovascular diseases (CVDs) worldwide, and CVDs remain among the leading causes of mortality and disability (Libby 2021). With an aging population and shifts in lifestyle habits, including unhealthy diets and physical inactivity, the global incidence of foam cell-related CVDs continues to rise (Psarros et al. 2012).

The accumulation of foam cells in atherosclerotic lesions is a key pathological feature of CVDs. These cells secrete pro-inflammatory cytokines and chemokines, which initiate and amplify local inflammatory responses, leading to plaque instability and, ultimately, acute coronary syndromes and stroke (Hansson et al. 2006). Current therapeutic strategies targeting foam cells mainly focus on lipid-lowering, anti-inflammatory interventions, and promoting cholesterol efflux (Guo et al. 2023; Libby 2024). However, major challenges remain, particularly in effectively modulating lipid metabolism and inflammation, as well as in the lack of specific therapeutic agents that directly target foam cells. Although statins and Proprotein Convertase Subtilisin/Kexin Type 9 (PCSK9) inhibitors significantly reduce low-density lipoprotein cholesterol (LDL-C) levels, a proportion of patients show poor responsiveness or develop adverse effects, and these treatments have limited effects on pre-existing foam cells (Dixon et al. 2019). In addition, although PPAR-γ agonists can regulate lipid metabolism and reduce inflammation, their long-term use may lead to metabolic disturbances and other side effects (Duan et al. 2008).

PPAR-γ plays a crucial role in the treatment of foam cell-related diseases by regulating lipid metabolism and inflammatory responses through multiple mechanisms, thereby reducing foam cell formation and accumulation (Franceschelli et al. 2023). As a nuclear receptor transcription factor predominantly expressed in adipose tissue, liver, and immune cells, PPAR-γ controls these processes by modulating gene expression (Chen et al. 2023). Upon activation, PPAR-γ upregulates the expression of key cholesterol transporters, such as ATP-binding cassette transporter A1 (ABCA1) and ATP-binding cassette transporter G1 (ABCG1), which are critical for reverse cholesterol transport. This promotes cholesterol efflux from macrophages and ultimately limits foam cell formation (Jiang et al. 2017). This mechanism is of great significance in the prevention and treatment of CVDs, including atherosclerosis. In summary, PPAR-γ is a pivotal regulator in foam cell-related pathology.

Perilla Fruit is a traditional Chinese medicinal herb with well-documented pharmacological effects, including lipid-lowering, anti-inflammatory, and antioxidant activities (Koonyosying et al. 2022; Paradee et al. 2021; Lee et al. 2013). It has been widely used in the treatment of atherosclerosis, hyperlipidemia, and other CVDs. Perilla Fruit contains numerous bioactive compounds such as unsaturated fatty acids, flavonoids, and polyphenols (Kaseke et al. 2020), which are known to inhibit foam cell formation and promote cholesterol efflux, thereby mitigating atherosclerotic plaque development.

In TCM, Perilla Fruit is often processed by stir-frying to enhance its therapeutic efficacy. This stir-frying process increases the concentration of certain active constituents, particularly polyphenols and flavonoids, enhancing its antioxidant, anti-inflammatory, and lipid-regulating effects (Luo yuqin et al. 2020). Moreover, stir-frying improves both the chemical stability and bioavailability of Perilla Fruit, making it more effective in clinical practice.

However, the pharmacological basis underlying the enhanced efficacy of stir-fried Perilla Fruit remains unclear. In this study, we investigated the changes in chemical composition induced by stir-frying and applied computational biology and TCM chemobiology approaches to explore how the active components of stir-fried Perilla Fruit modulate foam cell formation via PPAR-γ. This research provides mechanistic insights into the enhanced efficacy of processed Perilla Fruit and offers a scientific foundation for its clinical application.

## Materials and methods

### Chemicals and reagents

Luteolin (B20888) was purchased from Shanghai Yuanye. The Cell Counting Kit-8 (CCK-8; CK04) was purchased from Dojindo (Japan). The lactate dehydrogenase (LDH) cytotoxicity kit was purchased from Dojindo (Japan). Ox-LDL (YB-002) was purchased from Guangzhou Yiyuan. The Oil Red O staining kit (MAK194) was purchased from Sigma-Aldrich. Antibodies against ABCA1 (ab7360) and PPAR-γ (ab272718), and recombinant liver X receptor alpha (LXR-α; ab3585), were purchased from Abcam. GW9662 (HY-16578) was purchased from MedChemExpress (MCE).

Perilla Fruit were selected, with impurities removed, followed by thorough rinsing and drying. The processed fruit were then transferred into a preheated iron wok and subjected to slow heating at 150 °C over a moderate flame. Continuous stirring was maintained until the surface color darkened significantly, accompanied by the emission of popping sounds and the release of a characteristic aroma, at which point the heat source was terminated; this entire heating process was strictly controlled to last 6 min. Subsequently, the fruit were left in the wok to undergo further roasting utilizing residual heat for an additional 2 min, after which they were promptly removed and allowed to cool to ambient temperature.

### HPLC-QQQ-MS/MS

*Chromatographic conditions* For content determination and tissue distribution study, the HPLC-QQQ-MS/MS system was performed on an Agilent 1290 series HPLC system combined with an Agilent 6470 Triple Quadrupole Mass (Agilent Technologies, United States) equipped with an electrospray ionization (ESI) source.

*Chromatographic column* ACQUITY column (2.1 × 100 mm, 1.7 µm); Mobile phase: 0.1% formic acid aqueous solution (A), acetonitrile (B); Gradient elution, elution procedure: 0–1 min, 15–30 B%; 1–5 min, 30–98 B% flow rate: 0.3 mL/min, column temperature: 15 ℃; Injection volume: 5 µL.

#### Mass spectrometry conditions

*Ion source* electric spray ion source (ESI); Detection mode: Multi reaction ion detection (MRM); Scanning mode: positive and negative ion scanning mode; Gas Temperature: 300 ℃; Gas Flow: 7 L/min; Nebulizer pressure: 35 psi.

### Molecular docking

Automatic mining tools are used for molecular docking, connecting rigid proteins to flexible ligands. The chemical structure of luteolin was obtained from The Open Chemistry Database of the National Institutes of Health (NIH) in the United States (https://pubchem.ncbi.nlm.nih.gov) (Kim et al. 2021) PPAR-γ structure was obtained from the crystal structure database of proteins (http://www.rcsb.org) (Burley et al. 2017). The PDB ID is 1FM9. Before docking, proteins are prepared by removing crystal heteroatoms and water molecules from the crystal structure of the protein. Then, perform polarity hydrogenation and ionization on the protein. Finally, the protein and ligand structures were converted to PDBQT format using AutodockTools 1.5.6. Molecular docking simulations and free binding energy calculations were performed using AutodockTools VINA software, and visualizations were constructed using PyMOL.

### Cellular thermal shift assay (CETSA)

The CETSA experiment was conducted in the manner described earlier (Dai, et al. 2018; Jafari et al. 2014). In short, the soluble protein lysate of RAW264.7 cells was loaded into PCR tubes and treated with luteolin (10 μM) at room temperature for 2 h before CETSA thermal pulse. Heat the solution at the specified temperature (37–63 °C) for 3 min, and then use an Applied Biosystems, Cool at 4 °C for 3 min in USA. After centrifugation for 20 min (20,000 g, 4 °C), the soluble supernatant was subjected to Western blot analysis.

### Cell culture

RAW264.7 cells were purchased from the Ce ll Resource Centre of the Shanghai Institute of Life Sciences, Chinese Academy of Sciences, and were cultured in Dulbecco’s modified Eaglet medium (Corning, 10–017-CVR) with 10% fetal bovine serum (BI). RAW264.7 cells were incubated at 37 ℃ in a humidified atmosphere of 5% CO_2_ and 95% air.RAW264.7 cells were cultured in normal culture and the inhibitor group was incubated for 6 h with the addition of 1 μM GW9662 before establishing foam cells.

### Establishment of foam cell

RAW264.7 cells were cultured in 96-well white plate and maintained in a cell culture incubator with a constant temperature of 37 °C and a humidified atmosphere of 5% CO_2_ and 95% air for 24 h. Foam cell models were established by stimulating cells with 40, 60, 80, 100, and 120 µg/mL gradient ox-LDL for 24 h. Ox-LDL was the optimized stimulation concentration and adopted for subsequent experiments.

### Cell viability assay

Inoculate RAW264.7 cells that have grown to 80–90% confluence into a 96 well culture plate at a rate of 1 × 10^5^ cells/well, and use luteolin (0.1, 1, 10, 100μ mol/L) Four different solutions of μmol/L were treated for 24 h. After the cells were treated, 100 μL Dulbecco modified Eagle and 10% CCK-8 atherosclerosis solution (Biosharp, Hefei, China) were used to replace the culture medium, and the cells were incubated in darkness at 37 °C and 5% CO_2_ for 1 h. Measure the degree of hardening at 450 nm using a microplate reader and calculate the percentage of cell viability for the degree of hardening.

### LDH detection

RAW264.7 cells were seeded into a 96 well culture plate at a rate of 1 × 10^5^ cells/well, and treated with four different solutions of luteolin (0.1, 1, 10, 100 μmol/L) for 24 h. After the processing time, transfer 50 μL of RAW264.7 cell culture supernatant to a new 96 well blank culture plate and add 50 μL of cytotoxin ONETM reagent to each well for water hardening. Incubate at 37 °C in the dark with 5% CO_2_ for 15 min. Finally, add 25 μL of the stopping solution to each well and adjust the absorbance using an enzyme marker at 490 nM.

### Oil red O staining

RAW264.7 cells were stained with Oil Red O solution to visualize the accumulation of intracellular lipids. In short, RAW264.7 cells were washed 3 times with PBS and then fixed with 4% paraformaldehyde for 20 min. After washing with 60% isopropanol for 5 min, the cells were stained with ORO solution at room temperature for 20 min. Then, wash the cells with distilled water to remove excess dye, observe the lipid droplets stained red using an optical microscope, and take photos.

#### Quantification of total cholesterol

Add 100 μ L RAW264.7 cell lysate to each well, mix and shake for 10 min. Transfer the supernatant to a 1.5 mL centrifuge tube, centrifuge at 2000 g at room temperature, and let it stand for 5 min. After centrifugation, transfer the supernatant to a newly labeled 1.5 mL centrifuge tube. Prepare a cholesterol detection solution in a 4:1 ratio and add it to each group. The reaction time is 20 min at 37 °C.

#### Western-blot analysis

Use a mixture of RIPA: PMSF = 100:1 to lyse cells. Superhardening fragmentation water hardening carried out in an ice bath. Use the BCA protein assay kit to determine the concentration of hardened proteins. The same amount of protein was subjected to sodium dodecyl sulfate (SDS) polyacrylamide gel electrophoresis. The protein in the gel was transferred to the PVDF membrane, and 5% skimmed milk was used to block the water hardening of the membrane for 2 h, and PPAR-γ antibody was incubated at 4 °C overnight. Wash the membrane 5 times (5 min each time) with TBST, and then incubate with the corresponding secondary antibody at room temperature for 2 h.

#### Molecular dynamics

The molecular dynamics simulations were carried out with Desmond/Maestro noncommercial version 2022.1 as a molecular dynamic’s software (Phillips et al. 2020; Khan et al. 2022). TIP3P water molecules were added to the systems, which were then neutralized by 0.15 M NaCl solution. After minimization and relaxation of the system, the production simulation was performed for 100 ns in an isothermal-isobaric ensemble at 300 K and 1 bar. Trajectory coordinates were recorded every 100 ps. The molecular dynamics analysis was performed using Simulation Interaction Diagram from Desmond.

#### Statistical analysis

Data are expressed atherosclerosis mean ± SD. One-way ANOVA was performed using Prism 8.0.1 (Graphpad, San Diego, CA, USA) to determine the significance of the results. *p* < 0.05 was considered to be statistically significant.

## Result

### Luteolin is a key bioactive compound enriched by perilla fruit rocessing

To investigate the chemical changes induced by the processing of Perilla Fruit, both raw and stir-fried Perilla powder (500 mg each) were subjected to ultrasonic extraction with methanol. The supernatants were analyzed using HPLC-QQQ-MS/MS to quantify six representative compounds: danshensu, quercetin, caffeic acid, chlorogenic acid, luteolin, and protocatechualdehyde. The results revealed that the content of luteolin was significantly increased after stir-frying, suggesting that luteolin may be a key pharmacologically active ingredient enhanced by the processing of Perilla Fruit (Table 1).

**Table 1:** Content of six components in perilla fruit before and after processing (µg/g) (n = 3)

Compound	S1	S2
Danshensu	7457.41 ± 0.62	9491.81 ± 1.76
Quercetin	67.19 ± 1.02	69.31 ± 1.19
Chlorogenic acid	1795.04 ± 0.33	1340.73 ± 0.7
Caffeic acid	50.73 ± 1.63	47.93 ± 3.34
Luteolin	27,419.82 ± 0.21	31,238.62 ± 0.38
Protocatechualdehyde	592.47 ± 0.36	636.34 ± 1.02

*Note* S1: before processing; S2: After processing; “n” meaninjection time

### Identification of the safe and effective concentration range of luteolin

**Fig. 1:**
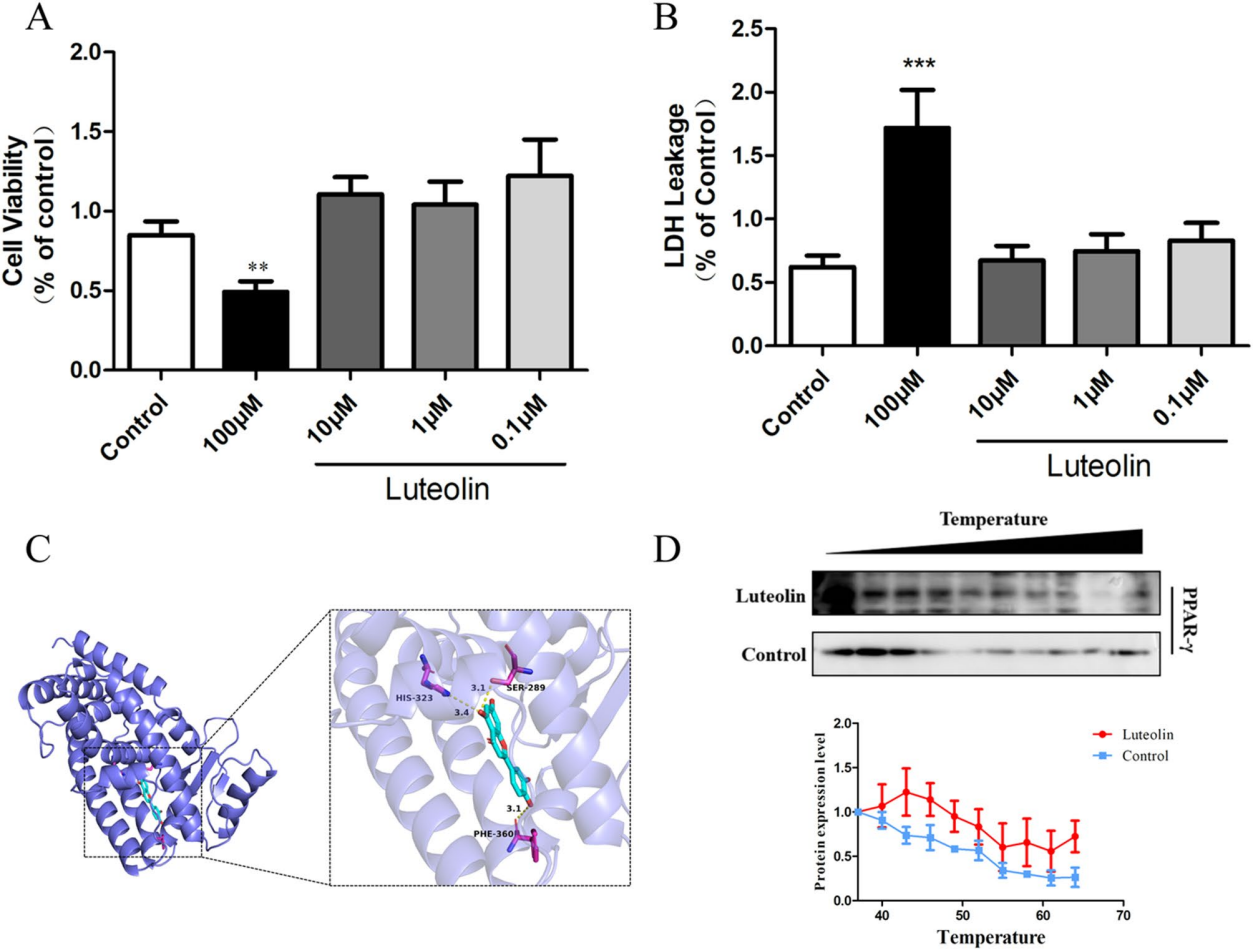
**A**: Screening of the optimal concentration of luteolin for RAW264.7 cell viability; **B**: Detection of the degree of damage to RAW264.7 cells caused by different concentrations of luteolin; **C**: Molecular docking of luteolin PPAR-γ complex; **D**: Under different stable environments, luteolin enhances the thermal stability of PPAR-γ protein

To evaluate the cytotoxicity and safe range of luteolin in vitro, RAW264.7 cells were treated with luteolin at concentrations of 0.1, 1, 10, and 100 μM for 24 h. Cell viability and membrane integrity were assessed using the CCK-8 and LDH release assays, respectively. The results showed that 100 μM luteolin significantly reduced cell viability and increased LDH leakage, while concentrations between 0.1 and 10 μM had no significant adverse effect on cell viability (Fig. 1A, B). Based on these findings, 10 μM was selected for subsequent experiments.

### Luteolin binds directly and stabilizes PPAR-γ

Molecular docking simulations demonstrated a stable interaction between luteolin and PPAR-γ, with a calculated binding energy of − 9.3 kcal/mol. Hydrogen bond analysis revealed that luteolin forms strong interactions with key residues SER289, HIS323, and PHE360 within the PPAR-γ ligand-binding domain.

To experimentally validate this interaction, a CETSA was performed using RAW264.7 cell lysates. The results indicated that treatment with 10 μM luteolin significantly increased the thermal stability of PPAR-γ protein compared to the DMSO control, supporting a direct and stabilizing interaction between luteolin and PPAR-γ (Fig. 1C, D).

### Luteolin promotes cholesterol efflux and inhibits foam cell formation via PPAR-γ activation

The effects of luteolin on foam cell lipid accumulation were evaluated using Oil Red O staining. Luteolin treatment markedly reduced intracellular lipid droplet formation, with the most pronounced effect observed at 10 μM. Consistently, total cholesterol levels in foam cells were significantly decreased following luteolin treatment.

**Fig. 2:**
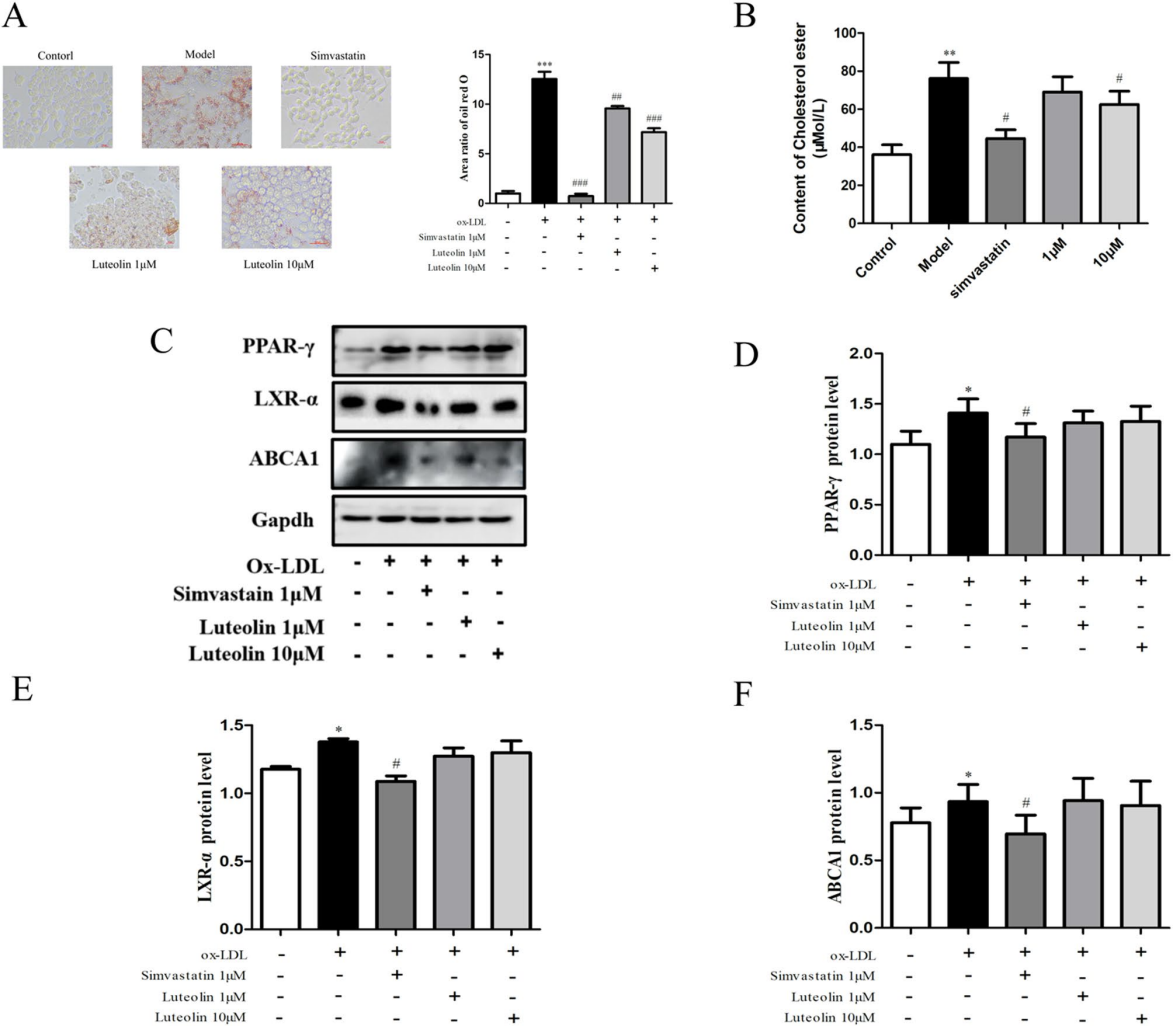
**A**: Luteolin inhibited the lipid droplets in foam cells; **B**: Luteolin can effectively reduce the cholesterol content in foam cells; **C**–**F**: Luteolin’s effect on PPAR-γ in foam cells, Regulation of LXR—α, ABCA1 protein expression

Western blot analysis revealed that the expression levels of PPAR-γ, LXR-α, and ABCA1 were significantly upregulated in foam cells, whereas luteolin intervention reversed their overexpression, suggesting a role in modulating cholesterol efflux pathways (Fig. 2).

**Fig. 3:**
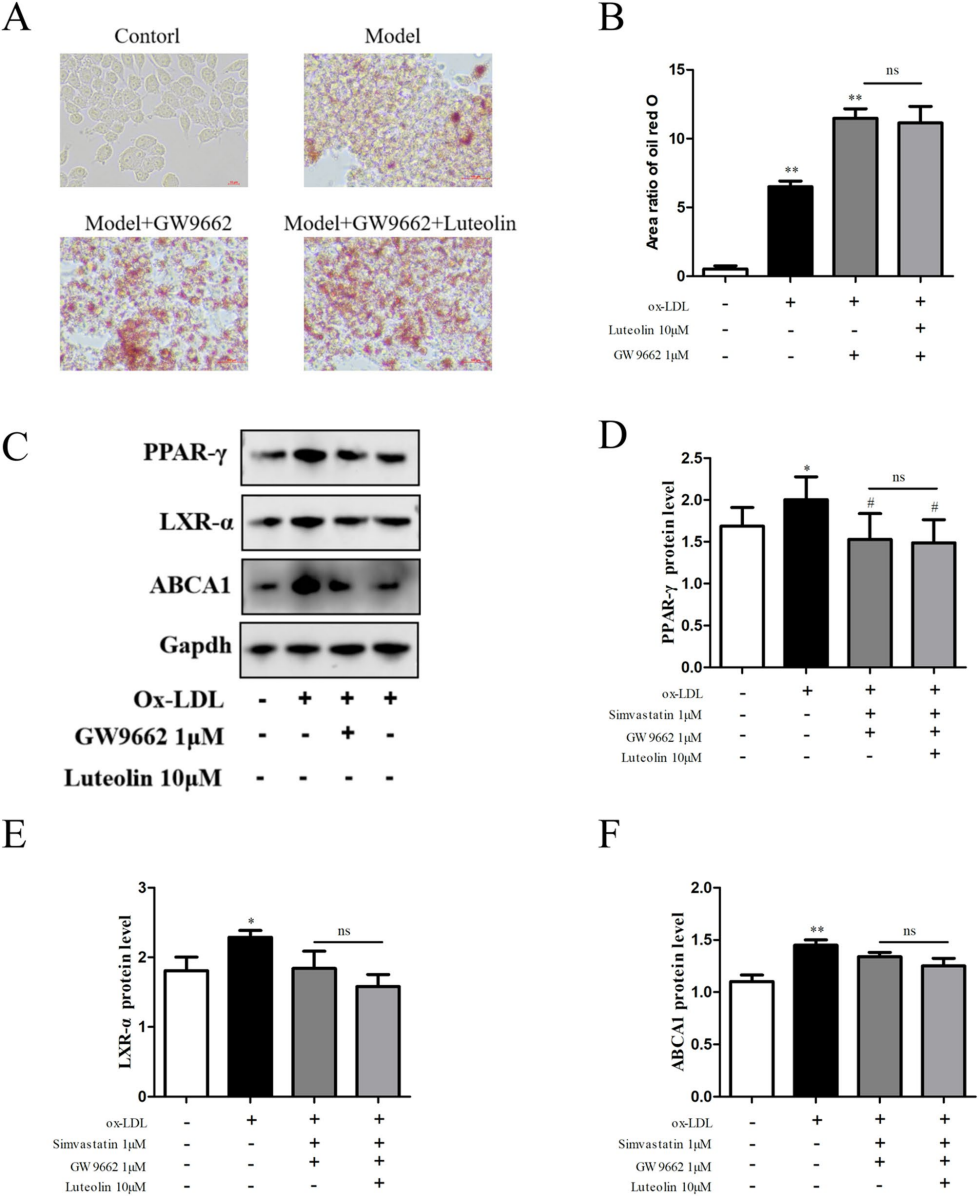
**A**, **B**: Under the intervention of GW9662, luteolin lost its inhibitory effect on lipid droplets in foam cells; **C**–**F**: Luteolin loses its effect on PPAR-γ in foam cells in GW9662 environment, Regulation of LXR -α, ABCA1 protein expression

To confirm whether the observed effects were mediated through PPAR-γ, the selective PPAR-γ antagonist GW9662 was used. Co-treatment with GW9662 abolished the lipid-lowering effects of luteolin, increased lipid droplet accumulation, and downregulated the expression of PPAR-γ, LXR-α, and ABCA1, indicating that the regulatory effects of luteolin on foam cells are dependent on PPAR-γ activation (Fig. 3).

### SER289, HIS323, PHE360, and TYR473 are potential key binding residues

To further assess the structural stability of the luteolin–PPAR-γ complex, a 100 ns molecular dynamics simulation was performed. The root mean square deviation (RMSD) analysis showed that the complex stabilized after approximately 40 ns, with protein RMSD fluctuating between 2.0–2.8 Å and ligand RMSD between 1.2–2.4 Å, indicating a robust and stable binding conformation.

**Fig. 4:**
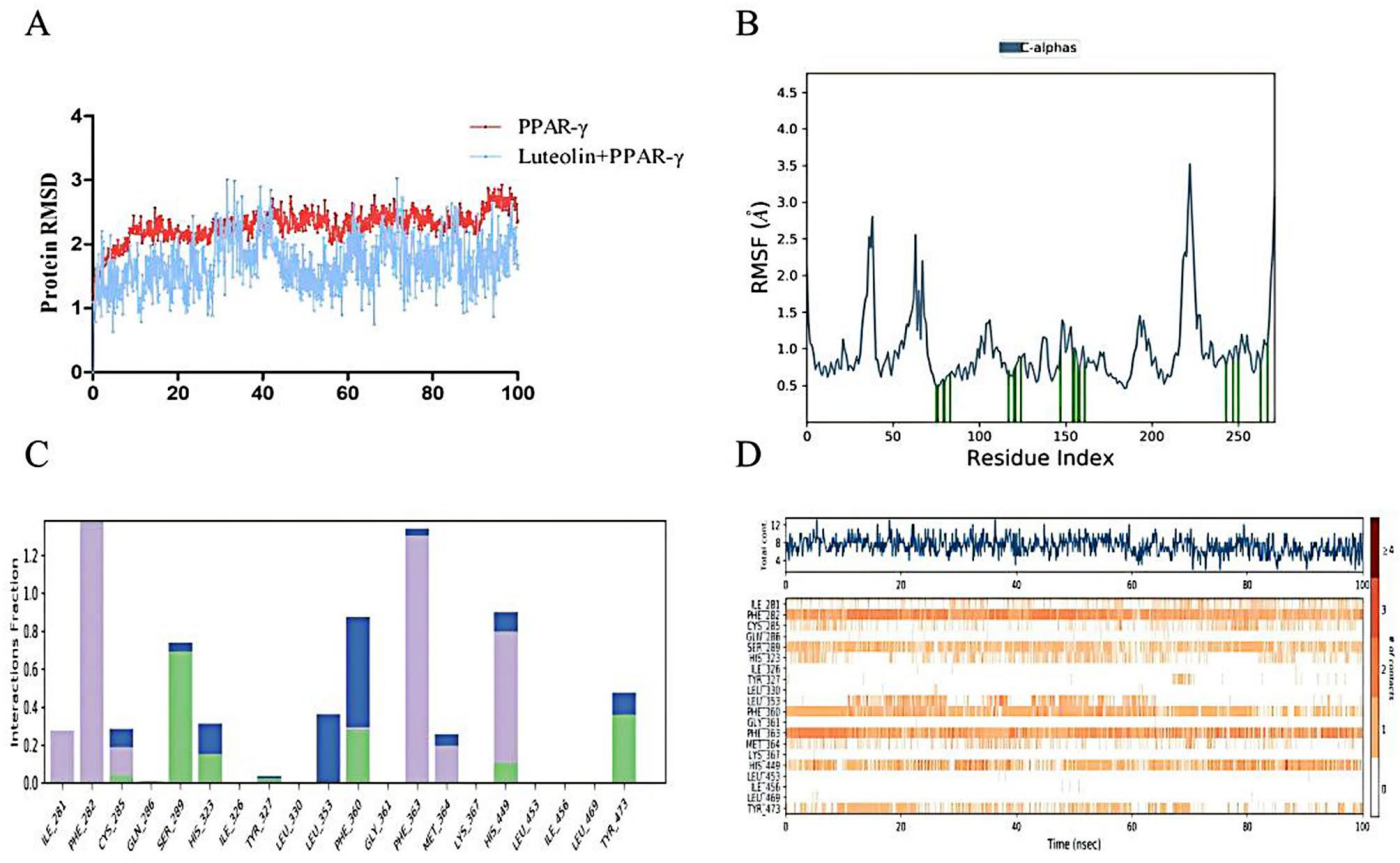
**A**: Molecular dynamics RMSD changes of the complex of luteolin and PPAR-γ within 100 ns; **B**: The RMSF position of the complex of luteolin and PPAR-γ; **C**: Protein Ligand contacts of the complex of luteolin and PPAR-γ, where green represents hydrogen bonds, purple represents hydrophobic bonds, and blue represents water bridges; **D**: Timeline representation of the interactions and contacts

Hydrogen bond trajectory analysis confirmed persistent interactions between luteolin and amino acid residues SER289, HIS323, PHE360, and TYR473, suggesting that these residues play critical roles in the recognition and stable binding of luteolin to PPAR-γ (Fig. 4).

## Discussion

Foam cells represent a hallmark pathological feature of atherosclerotic lesions. They arise when macrophages internalize oxidized low-density lipoprotein and accumulate within the arterial wall, contributing directly to the formation and progression of atherosclerotic plaques (Ma et al. 2024). Far from being passive lipid reservoirs, foam cells actively participate in the atherogenic process and significantly influence the pathophysiology of CVDs. The formation of foam cells begins with macrophage uptake of oxLDL via surface-expressed scavenger receptors such as scavenger receptor A (SR-A) and CD36 (Lin et al. 2012; Ooi et al. 2021). This lipid internalization leads to intracellular cholesterol accumulation and subsequent transformation into foam cells. Concomitantly, these cells secrete various pro-inflammatory cytokines and chemokines, including Tumour necrosis factor-α (TNF-α), Interleukin-1β (IL-1β), and monocyte chemotactic protein-1 (MCP-1), thereby exacerbating local inflammation and disease progression (Li et al. 2022; Silva et al. 2009).

Current therapeutic strategies for foam cell-associated disorders predominantly focus on lipid-lowering, anti-inflammatory, and cholesterol efflux-promoting approaches (Opazo-Ríos et al. 2020; Choudhury et al. 2005). Lipid-lowering agents such as statins and PCSK9 inhibitors reduce circulating lipid levels and oxLDL formation, thereby decreasing its uptake by macrophages (Wu and Ballantyne et al. 2017). Anti-inflammatory therapies aim to suppress the secretion of inflammatory mediators from foam cells, thereby alleviating vascular inflammation. Promoting cholesterol efflux involves the activation of nuclear receptors such as peroxisome proliferator-activated receptor gamma (PPAR-γ) and liver X receptor alpha (LXR-α), which upregulate ATP-binding cassette transporters ABCA1 and ABCG1, facilitating cholesterol removal from foam cells (Marasinghe et al. 2024).

Despite their clinical utility, these strategies are often limited by efficacy and safety concerns. Statins and PCSK9 inhibitors may cause adverse effects in some patients, including myopathy and hepatic dysfunction, and are not uniformly effective across patient populations (Feingold 2024). Long-term use of anti-inflammatory drugs can result in immunosuppression and an increased risk of infections (Al-Khalili A et al. 2018). Moreover, while PPAR-γ and LXR-α agonists effectively promote cholesterol efflux, their use may induce metabolic disturbances and off-target effects, underscoring the need for improved specificity and safety profiles. Overall, current treatment modalities exhibit limited effectiveness against established foam cells, and more refined therapeutic approaches are urgently needed.

The observed changes in luteolin and chlorogenic acid content following stir-frying of Perilla Fruit may be linked to shifts in their biosynthesis pathways. Chlorogenic acid, a phenylpropanoid derivative synthesized via the shikimate pathway, is prone to thermal degradation during high-temperature processing. Stir-frying likely accelerates its hydrolysis or oxidation, as phenolic esters like chlorogenic acid are thermally labile-this could explain the 25% reduction in its content (S1: 1795.04 µg/g vs. S2: 1340.73 µg/g). In contrast, luteolin, a flavonoid derived from the phenylpropanoid-flavonoid pathway, may accumulate due to thermal activation of key biosynthetic enzymes (e.g., chalcone synthase, flavonol synthase) during processing. Stir-frying might also disrupt plant cell wall structures, enhancing the extraction efficiency of luteolin from intracellular compartments, contributing to its 14% increase (S1: 27,419.82 µg/g vs. S2: 31,238.62 µg/g). Notably, these changes could reflect a metabolic trade-off: reduced chlorogenic acid may free up precursor molecules (e.g., cinnamic acid) that are redirected toward flavonoid biosynthesis, favoring luteolin accumulation. However, further studies targeting gene expression of pathway-specific enzymesin processed Perilla Fruit are needed to confirm these regulatory mechanisms. Such insights would deepen understanding of how TCM processing modulates phytochemical profiles to enhance therapeutic efficacy.

TCM offers unique advantages in addressing the shortcomings of current therapeutic options for foam cell-related disorders (Zhang et al. 2020). With its multi-target, multi-pathway pharmacological actions, TCM can comprehensively modulate lipid metabolism and inflammatory responses. Compared to conventional drugs, TCM formulations exhibit fewer side effects, improved long-term safety, and greater individual adaptability, largely due to synergistic interactions among their active constituents. These features position TCM as a valuable complementary or alternative strategy in cardiovascular disease management.Perilla Fruit.

Among TCM strategies, the processing (Paozhi) of herbal materials plays a pivotal role in enhancing efficacy. Specifically, stir-frying has been shown to enrich bioactive components such as polyphenols and flavonoids, enhancing its antioxidant, anti-inflammatory, and lipid-regulatory properties. These chemical changes are associated with improved efficacy in preventing and treating CVDs (Pan et al. 2020; Wang et al. 2021; Masuda et al. 2018). However, the pharmacological basis underlying these enhanced effects has remained poorly defined. Our study addresses this knowledge gap by providing mechanistic insights into the processing-induced pharmacological enhancement.

Luteolin, a naturally occurring flavonoid, has recently attracted attention for its interaction with PPAR-γ (He, et al. 2023). Our data demonstrate that luteolin forms a stable complex with PPAR-γ, exhibiting a binding free energy of − 9.3 kcal/mol. This interaction is reinforced by hydrogen bonds with key residues SER289, HIS323, and PHE360, contributing to structural stability. Additionally, CETSA confirmed that luteolin markedly increases the thermal stability of PPAR-γ in RAW264.7 cell lysates, indicating a direct and functionally relevant binding interaction. The significance of this interaction lies in PPAR-γ’s role as a central transcriptional regulator of lipid metabolism and inflammation. By activating PPAR-γ, luteolin enhances the expression of downstream targets such as LXR-α and ABCA1, which are essential for promoting cholesterol efflux and restoring lipid homeostasis. Functionally, luteolin reduces foam cell formation, attenuates plaque progression, and contributes to plaque stabilization-thereby potentially lowering the risk of adverse cardiovascular events (Zheng et al. 2021).

Moreover, Luteolin, as a key bioactive flavonoid enriched in processed Perilla Fruit, its bioavailability and pharmacokinetics in humans are critical for translating preclinical findings to clinical applications. Existing studies have shown that luteolin exhibits low oral bioavailability due to its poor water solubility, extensive first-pass metabolism, and intestinal efflux mediated by transporters such as P-glycoprotein. After oral administration, luteolin is primarily absorbed in the small intestine, but its absorption rate is limited by its hydrophobicity. However, the stir-frying processing of Perilla Fruit, which enhances luteolin content, may also influence its bioavailability indirectly. For instance, thermal processing can alter the matrix structure of Perilla Fruit, potentially facilitating the release of luteolin during digestion and improving its dissolution rate in the gastrointestinal tract.

In terms of pharmacokinetics, luteolin undergoes extensive metabolism in humans, including glucuronidation and sulfation in the liver and intestines, forming water-soluble conjugates that are primarily excreted via urine and bile. The plasma half-life of luteolin is relatively short, which may require multiple administrations to maintain effective concentrations. Notably, the coexistence of other bioactive components in processed Perilla Fruit, such as unsaturated fatty acids and polyphenols, might affect luteolin’s pharmacokinetic profile. For example, certain fatty acids can enhance the solubility of luteolin in lipid-based formulations, promoting its absorption, while other polyphenols may inhibit metabolic enzymes like UDP-glucuronosyltransferases, reducing luteolin’s conjugation and extending its systemic exposure. Despite these insights, further studies are needed to specifically investigate the bioavailability and pharmacokinetics of luteolin from stir-fried Perilla Fruit in humans. Such research should consider factors like dosage forms, dietary interactions, and individual variations in metabolism to determine the optimal clinical administration strategy for maximizing its anti-atherogenic efficacy.

In summary, our findings provide compelling evidence that luteolin acts as a PPAR-γ agonist and mediates anti-atherogenic effects through modulation of lipid metabolism and inflammation. This work lays a theoretical and mechanistic foundation for the development of novel anti-atherosclerosis agents based on luteolin or its structural analogs. Moreover, elucidation of the PPAR-γ-luteolin interaction expands our understanding of flavonoids as functional modulators of nuclear receptors. Finally, this study offers scientific support for the rationality and efficacy of traditional Chinese medicinal processing, highlighting its potential to optimize the pharmacological properties of herbal medicines through modern biological validation.

The clinical relevance of these findings lies in their potential to bridge traditional herbal practice with modern cardiovascular therapy. Stir-fried Perilla Fruit, as a widely used TCM with enhanced luteolin content, could serve as a natural source for developing novel PPAR-γ-targeted interventions. For translational research, immediate next steps include evaluating the in vivo efficacy of stir-fried Perilla extracts in atherosclerosis animal models (e.g., ApoE⁻/⁻ mice) to confirm lipid-lowering and plaque-stabilizing effects. Subsequent phase I/II clinical trials should focus on determining optimal dosages of stir-fried Perilla preparations, assessing their safety profile in healthy volunteers, and verifying reductions in circulating pro-atherogenic markers (e.g., ox-LDL, inflammatory cytokines) in patients with hyperlipidemia. Additionally, exploring combination therapies with existing lipid-lowering agents (e.g., low-dose statins) may synergistically enhance efficacy while minimizing side effects, offering a promising avenue for clinical translation. These steps are critical to validating the therapeutic potential of processed Perilla Fruit in human atherosclerotic disease.

However, it is important to acknowledge the limitations of the present study. All experimental data were obtained from in vitro models using RAW264.7 macrophage cells, which, although widely used for foam cell research, do not fully replicate the complexity of atherosclerotic processes in vivo. Cellular responses in vitro may differ significantly from those in animal models or human subjects due to differences in metabolism, immune regulation, and pharmacokinetics. Furthermore, the lack of in vivo or clinical validation limits the direct translational applicability of our findings. Future studies should include in vivo experiments in relevant atherosclerosis models and eventually clinical trials to fully evaluate the efficacy, safety, and pharmacodynamic behavior of luteolin and processed Perilla Fruit in the context of cardiovascular disease.

## Supplementary information

Additional file1 (334 kb)

## Data Availability

Data sharing is not applicable to this article as no datasets were generated or analysed during the current study.

## Abbreviations

ABCA1: ATP-binding cassette transporter A1
ABCG1: ATP-binding cassette transporter G1
CCK-8: Cell counting Kit-8
CETSA: Cellular thermal shift assay
CVDs: Cardiovascular diseases
IL-1β: Interleukin-1β
LDL-C: Low-density lipoprotein cholesterol
LDH: Lactate dehydrogenase
LXR-α: Recombinant liver X receptor alpha
MCP-1: Monocyte chemotactic protein-1
ox-LDL: Oxidized low-density lipoprotein
PCSK9: Proprotein convertase subtilisin/kexin type 9
PPAR-γ: Peroxisome proliferator-activated receptor gamma
SR-A: Scavenger receptor A
TCM: Traditional chinese medicine
TNF-α: Tumour necrosis factor-α
