# Supplementary material for: Traditional processing unlocks anti-atherogenic potential of perilla fruit via PPAR-γ activation by luteolin
Source: Bioresour Bioprocess. 2025 Nov 3;12(1):124. doi: 10.1186/s40643-025-00957-7 (PMC12583437; doi:10.1186/s40643-025-00957-7)

CETSA

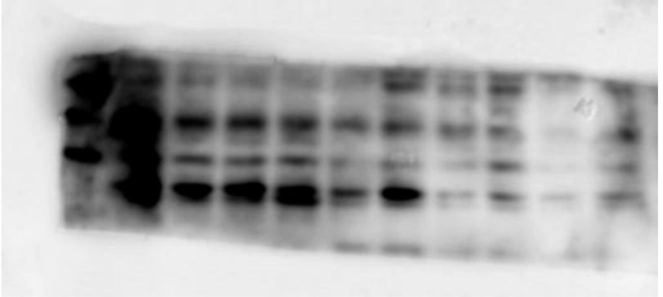

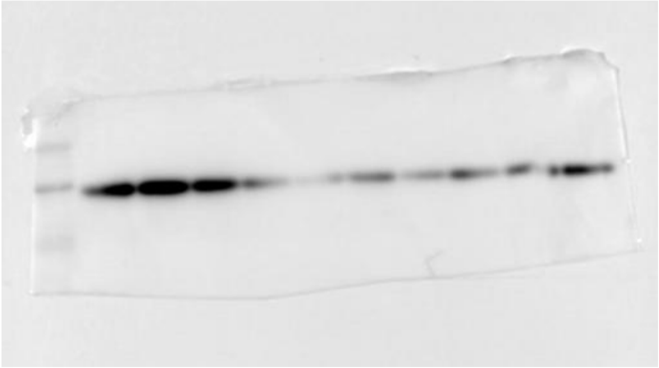

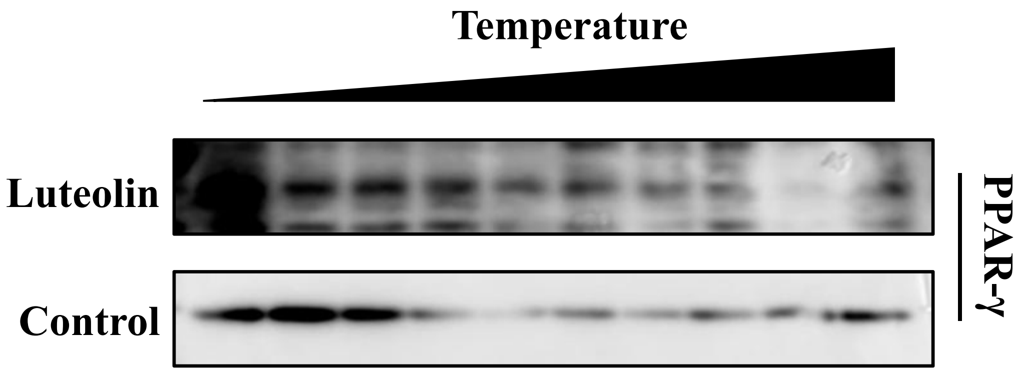

PPAR-γ

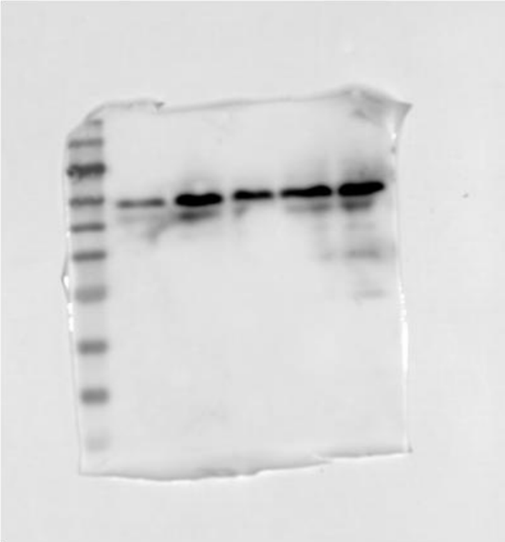

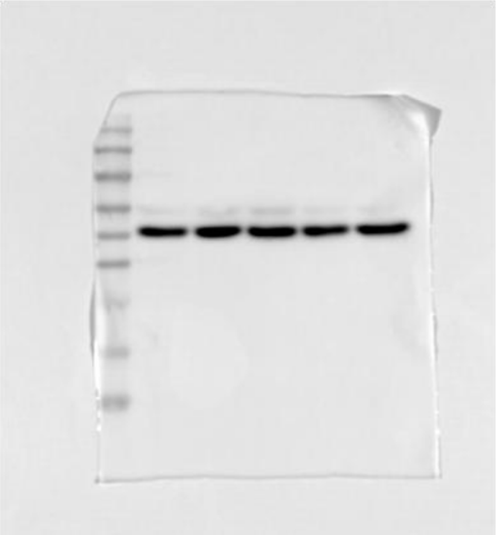

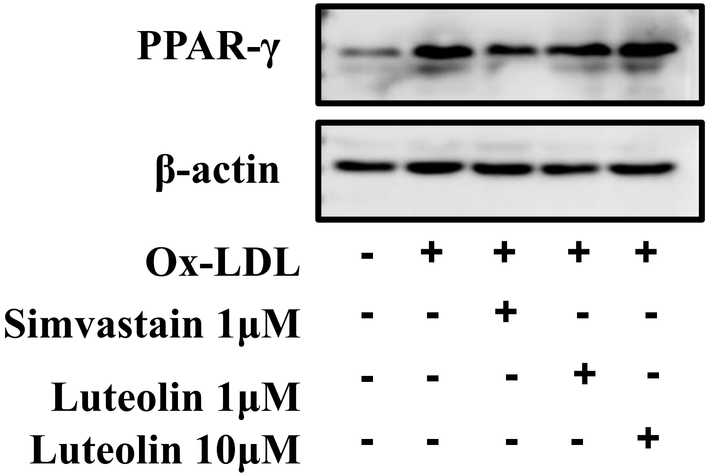
PPAR-γ
β-actin
Ox-LDL - + + + +
Simvastain 1μM - - + - -
Luteolin 1μM - - - + -
Luteolin 10μM - - - - +

LXR-α

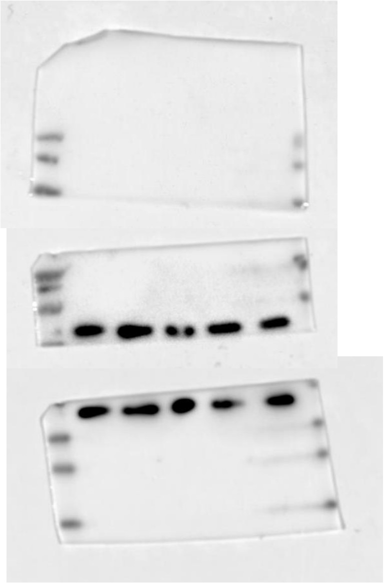

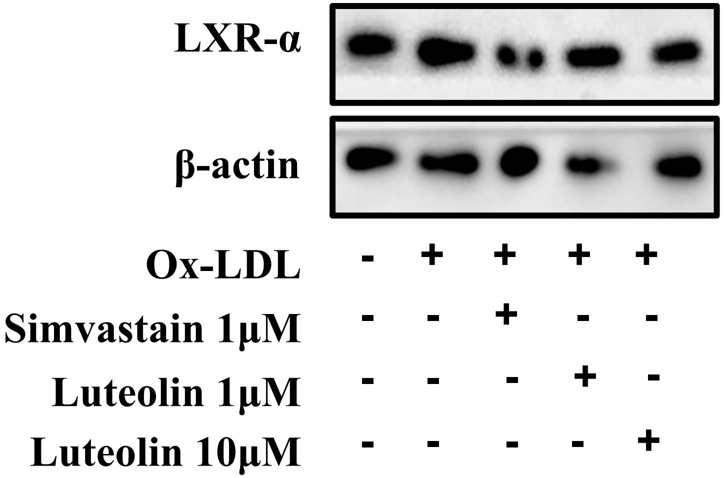
LXR-α
β-actin
Ox-LDL - + + + +
Simvastain 1μM - - + - -
Luteolin 1μM - - - + -
Luteolin 10μM - - - - +

ABCA1

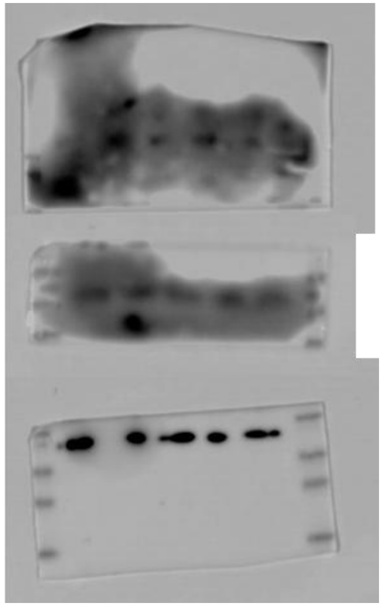

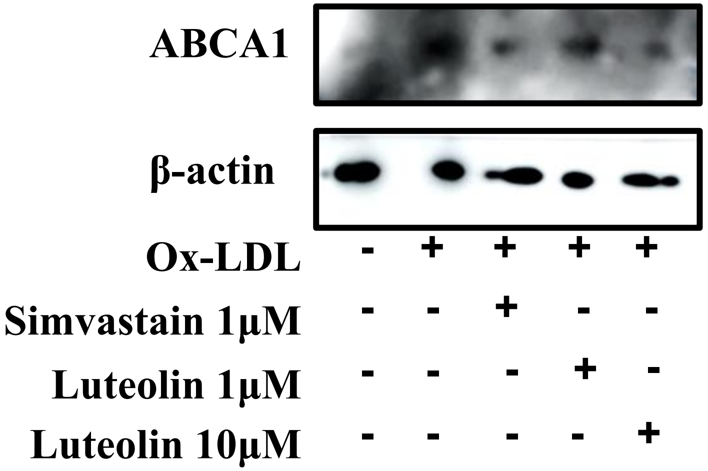
ABCA1
β-actin
Ox-LDL - + + + +
Simvastain 1μM - - + - -
Luteolin 1μM - - - + -
Luteolin 10μM - - - - +

PPAR-γ

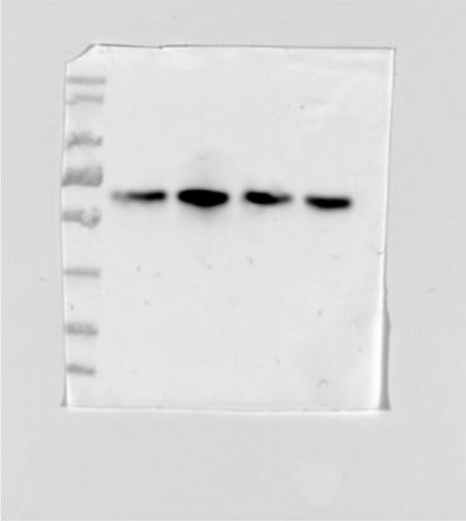

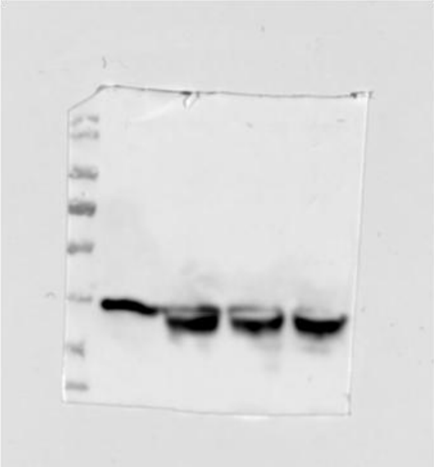

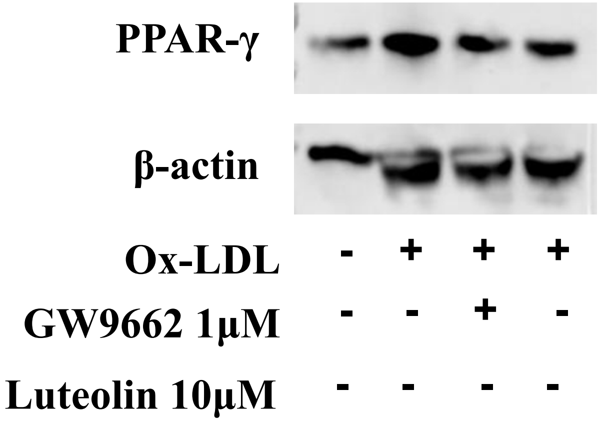

LXR-α

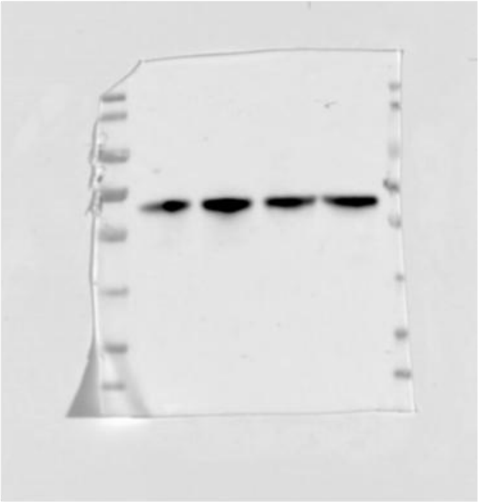

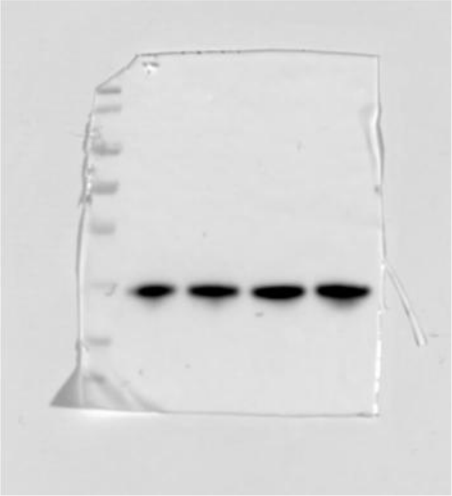

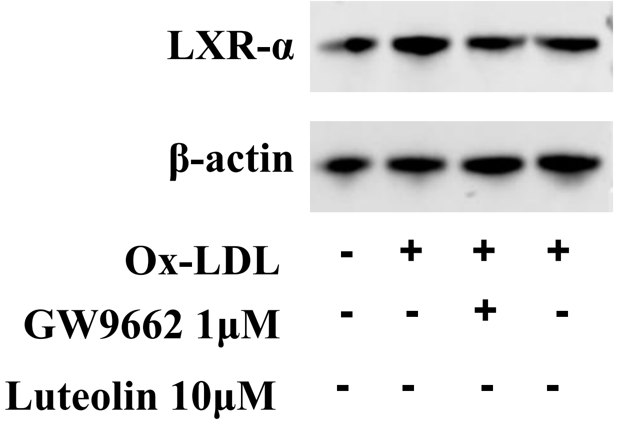

# ABCA1

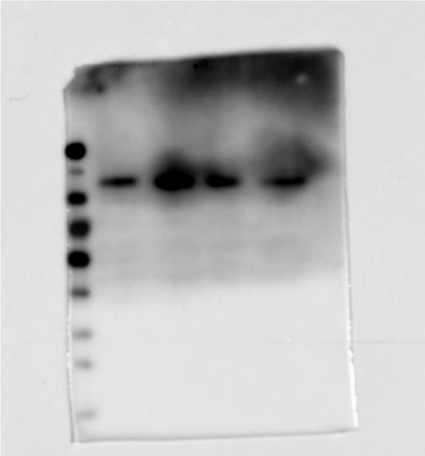

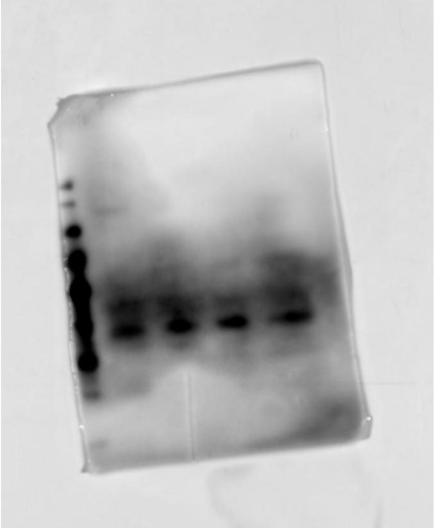

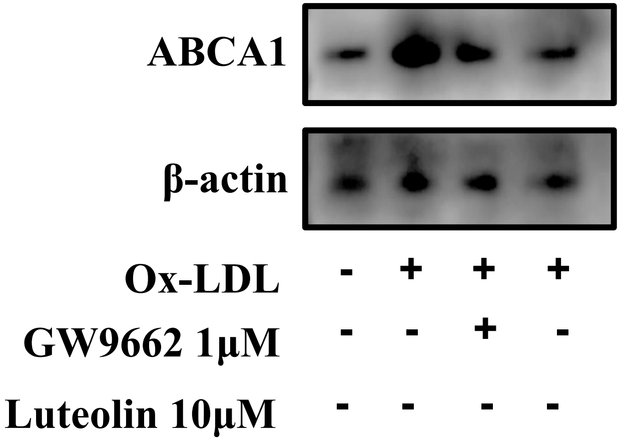

Supplement: Supplementary file 1 — Additional file1 (334 kb) [file 40643_2025_957_MOESM1_ESM.pdf]
